# The impact of fertilization on the health of paddy soil: pathways and prospects for fertility regulation based on microbial communities

**DOI:** 10.1007/s44297-026-00078-3

**Published:** 2026-06-09

**Authors:** Hongyang Xu, Aiping Shu, Shurong Gan, Xiao Han, Xinyi Zhang, Wenxue Zhang, Zengbing Liu, Jinbiao Ma, Wenchong Shi, Zheng Gao

**Affiliations:** 1 Institute of Soil and Fertilizer & Resource and Environment, China/National Engineering and Technology Research Center for Red Soil Improvement/Key Laboratory of Crop Ecophysiology and Farming System for the Middle and Lower Reaches of the Yangtze River, Ministry of Agriculture and Rural Affairs/Laboratory of Acidiffed Soil Amelioration and Utilization/National Agricultural Experimental Station for Agricultural Environment, Yichun/Jiangxi Provincial Key Laboratory of Arable Land Improvement and Quality Enhancement, Nanchang, Jiangxi China; 2 State Key Laboratory of Wheat Improvement, College of Life Sciences, Shandong Agricultural University, Tai’an, Shandong China; 3 Rice Research Institute of Jiangxi Academy of Agricultural Sciences, Nanchang, Jiangxi China

**Keywords:** Rice, Paddy soil, Microorganism, Fertilizer, SOC storage

## Abstract

Fertilization is crucial for enhancing soil fertility and crop yields, primarily by modifying soil physicochemical properties and microbial communities, which subsequently influence soil carbon storage. Due to the unique flooded conditions of rice cultivation, paddy soils exhibit distinct microbial compositions and carbon storage mechanisms compared to upland soils. However, indiscriminate fertilization, without a thorough understanding of the underlying soil carbon pool storage and turnover mechanisms, not only yields suboptimal results but also poses environmental risks. Currently, a comprehensive literature review exploring the impacts of fertilization on crop growth and soil fertility accumulation mechanisms within rice cultivation systems remains lacking. This review summarizes the nutrient characteristics and turnover mechanisms in paddy soils, revealing slower organic matter turnover and stable soil organic carbon due to the unique paddy soil anaerobic environment. Microbial mineralization and organic matter accumulation vary with the planting cycle. We discuss the impacts of fertilization on soil fertility and microbial communities, highlighting the superior and more environmentally friendly effects of organic fertilizers. Importantly, a saturation threshold exists for soil carbon storage; exceeding this limit renders fertilization ineffective. Appropriate fertilizer application positively impacts microbial communities by modifying soil pH and physicochemical properties. Furthermore, this paper elaborates on the mechanisms of microbial-mediated carbon sequestration and its influencing factors. Future research leveraging a clear understanding of paddy soil nutrient mechanisms should integrate synthetic biology, materials engineering, and data-driven intelligent decision-making systems. This approach promises to be an effective pathway toward achieving intelligent, green, and high-yielding rice cultivation.

## Introduction

Rice (*Oryza sativa* L.), a member of the Poaceae family, originated in China, India, and other Asian regions. As a pivotal cereal crop globally [1], its yield potential is predominantly governed by two agronomic determinants: (i) edaphic conditions characterized by high fertility, optimal porosity, and elevated organic matter content and (ii) intrinsic nutrient use efficiency. In contemporary agricultural systems, fertilization is a critical management intervention to augment productivity. Fertilizers are classified into inorganic (synthetic) and organic variants based on their provenance and physicochemical properties. Inorganic fertilizers are synthesized or mineral-derived compounds existing primarily as inorganic salts, with their principal components being single or compound mineral nutrients. In contrast, organic fertilizers are materials produced from the fermentation and maturation of animal and plant residues, excreta, and other organic matter. Their primary constituents include organic matter, essential nutrients, humic substances (e.g., fulvic acid and humic acid), and biologically derived structural components of biological origin. Inorganic fertilizers, favored for their rapid nutrient mobilization and high commercial scalability, undergo dynamic solubility transitions in soil matrices due to environmental variables (e.g., pH, moisture), thereby modulating mineral nutrient availability. Long-term excessive application of chemical fertilizers may reduce soil fertility, alter microbial abundance and composition, and cause various environmental issues [2]. Soil organic matter (SOM) refers to all organic matter present in the soil and is one of the important indicators for measuring soil fertility. In recent years, research on organic fertilizers has received increasing attention. Organic fertilizers enhance crop growth by (i) direct organic carbon input, (ii) soil structural stabilization via aggregate formation mediated by microbial activity, and (iii) microbially facilitated SOM mineralization that liberates plant-available nutrients. However, their slower nutrient release limits widespread adoption in intensive farming systems [3]. Recently, the combined application of organic and inorganic fertilizers has been proposed as a potential strategy to ensure sustainable soil productivity while maintaining environmental quality [4].

Soil microorganisms play a crucial role in the transformation of SOM. As key contributors to SOM storage, microbial communities convert decomposed carbon into recalcitrant metabolic products, such as polysaccharides and lipids, through intracellular biochemical processes. Additionally, they facilitate SOM stabilization via the secretion of extracellular polymeric substances (EPS), which promote the binding of metabolized organic compounds to mineral surfaces, forming mineral-associated organic matter (MAOM, a stable carbon pool). MAOM refers to the complex formed by the combination of organic matter and soil minerals through physical and chemical reactions and is an important component of soil organic carbon. This transformation process is recognized as a microbially mediated direct SOM storage mechanism [5]. In addition to this direct effect, soil microorganisms indirectly promote SOM accumulation through various pathways that mediate the transformation of plant litter and animal residues into particulate organic matter (POM, a labile carbon pool). POM primarily consists of partially decomposed plant detritus, microbial necromass, and organo-mineral microaggregates. Due to its high lability and rapid turnover, POM represents a labile fraction of soil organic matter that plays a key role in short-term carbon cycling and nutrient dynamics.

**Fig. 1:**
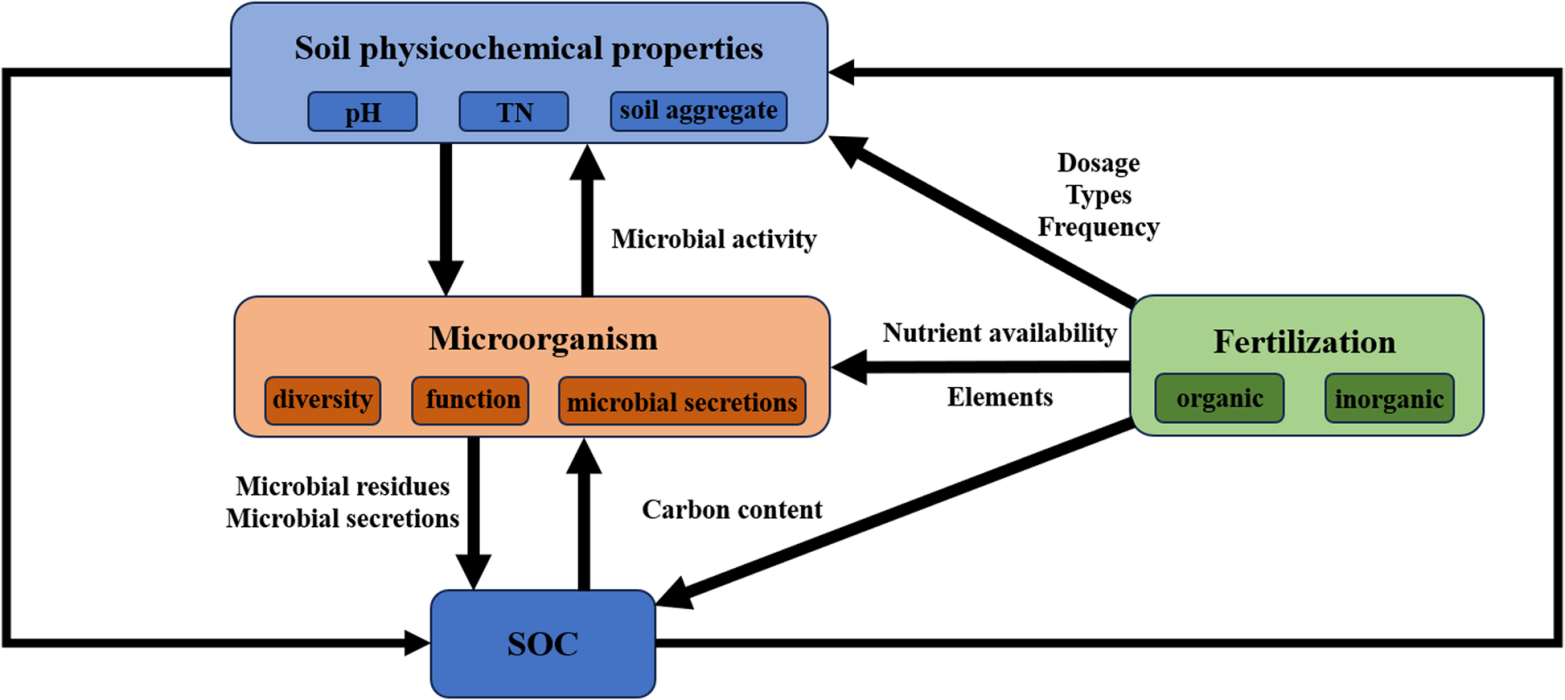
Conceptual framework between fertilization, soil physicochemical properties, and microorganisms. SOC: soil organic carbon; TN: total nitrogen

Paddy soils, formed under long-term rice cultivation through the combined effects of anthropogenic hydromorphic management and natural pedogenic processes, exhibit distinct profile configurations. Compared to upland soils, paddy soils exhibit higher organic matter content but with a simpler composition. Researchers generally believe that the flooding cultivation mode of paddy soil enhances the accumulation of soil organic carbon (SOC, the carbon-containing portion of SOM). This is primarily attributed to waterlogging-induced anaerobic conditions and the absence of alternative terminal electron acceptors, preventing effective organic matter oxidation and decomposition at low Eh values [6]. However, soil carbon sequestration capacity is not infinite; rather, it is constrained by a physicochemical upper limit known as the “soil carbon saturation threshold” [7], governed by the finite surface area of the mineral matrix. This threshold is often quantitatively characterized through indicators, for instance, the MAOC (the carbon-containing portion of MAOM), the relationship between SOC and fine silt content, or the “carbon saturation deficit” (CSD) [8, 9]. CSD is defined as the difference or ratio between the soil’s current stable carbon stock and its theoretical maximum stable capacity. The higher CSD values thus imply a larger remaining sequestration capacity. Conversely, a lower CSD suggests that the soil is approaching saturation, where additional organic C preferentially partitions into rapidly cycling POM rather than stable fractions, thereby lowering the stabilization efficiency [10]. This soil carbon saturation threshold varies significantly depending on the soil type and fertilization regime. Typically, soils with higher clay content, larger specific surface area, and distinct iron-aluminum oxide compositions possess a greater potential for forming MAOM and a higher carbon saturation capacity [10–12]. In contrast, coarse-textured soils are more prone to reaching their stable carbon pool capacity limits sooner [13]. Consequently, soils approaching this saturation point often exhibit reduced sequestration efficiency, suggesting that indiscriminate fertilization may result in diminishing returns regarding carbon storage. Although extensive literature has examined the effects of fertilization on crop growth and soil fertility, existing studies have often failed to systematically integrate fertilization strategies with the unique microbial communities and specific carbon saturation mechanisms in paddy soils. This review was conducted via literature searches in databases including PubMed, Dota Scholar, and Web of Science, covering the period from 2020 to 2025 (with some references from 2015 to 2020). Preliminary screening utilized keywords such as “rice”, “paddy”, “microbe”, “fertilization”, “soil”, and “nutrition”, yielding approximately 200 relevant articles. Subsequent inclusion criteria incorporated terms such as “SOM”, “organic fertilizer”, “inorganic fertilizer”, “carbon stock”, and “agricultural practices”, which narrowed the selection to approximately 180 relevant literature sources. In light of this, based on the functional differences between paddy soil and upland soil, this paper will focus on exploring the effects of organic fertilizer application on the microbial community and soil fertility in paddy soil. It aims to clarify the carbon storage mechanism in paddy soil, dominated by microorganisms, elucidate the limiting factors influencing soil carbon storage in paddy fields, attempt to summarize methods for achieving high fertilizer utilization efficiency, and construct a clear conceptual framework that elucidates the dynamic interrelationships between fertilization, soil properties, and microbial processes within paddy ecosystems (Fig. 1). This endeavors to provide a theoretical basis and evidence for achieving high rice yields in the future.

## The effects of microorganisms on nutrient transformation in paddy soils and nutrient uptake in rice

The composition of microorganisms and the physiological activities and structure of rice roots are the main driving forces for nutrient transformation in paddy soils and nutrient uptake by rice. This chapter reviews three aspects of microorganism-mediated nutrient transformation in paddy soils and the mechanisms promoting root nutrient uptake: (i) Microorganism-mediated mineralization of organic matter in paddy soil. (ii) Microorganism-mediated accumulation of organic matter in paddy soil. (iii) The impact of soil microorganisms on nutrient uptake by rice roots.

### Characteristics of paddy soil nutrients

Paddy soils possess unique characteristics compared to other soil types. Their long-term waterlogged condition results in significant differences in physicochemical properties compared to upland soils. The availability of nutrients (e.g., N, P, K, Fe, Si) and organic matter content significantly impact rice yield and quality in paddy soils.

Due to waterlogged conditions, redox reactions, microbial activity, and various environmental factors, paddy soils are often subject to deficiencies in nitrogen (N), phosphorus (P), and potassium (K). Microbially mediated mineralization facilitates the transformation of organic nitrogen into ammonium (NH_4_^+^). Subsequently, nitrification converts ammonium into nitrate (NO_3_^–^), which is readily assimilated by plants and microorganisms. Under the anaerobic conditions typical of paddy fields, denitrifying bacteria proliferate and mediate the reduction of nitrate (NO_3_^−^, derived from nitrification) to gaseous nitrogen (N_2_) through denitrification. This microbial process represents a major pathway of nitrogen loss, occurring primarily through N_2_ emissions, with additional losses via NO_3_^−^ leaching and runoff [14]. In flooded acidic soils, P is readily solubilized and mobilized, thereby enhancing P mineralization and increasing the pool of plant-available P [15]. However, excessive fertilizer application frequently results in asynchrony between P availability and crop demand, leading to substantial P losses through runoff and consequent soil P depletion [16]. In agricultural practice, these scenarios typically require the implementation of multiple strategies, including (i) promoting soil microbial communities involved in nitrogen cycling, (ii) incorporating crop straw residues, (iii) applying wood ash as an amendment, and (iv) supplementing with potassium and phosphate fertilizers. Although silicon (Si) content is typically high in paddy soils, its bioavailability is limited due to low solubility, existing predominantly as monomeric silicic acid [Si(OH)_4_] in the soil solution. Si can be adsorbed onto iron (Fe) and aluminum (Al) oxide colloids and may form complexes with ferric hydroxide [Fe(OH)_3_]. Flooding conditions enhance the reductive dissolution of these compounds, thereby increasing Si availability to meet rice growth requirements.

The organic matter pool in paddy soils comprises humus, substantial quantities of undecomposed organic material, microbial residues, and colloidal substances. Notably, the humic acid/fulvic acid (H/F) ratio, aromaticity, and molecular weight of paddy soil organic matter are consistently lower than those in other soil types, resulting in a reduced turnover rate of SOM. Under hypoxia and low redox potential conditions, water-soluble metabolic intermediates —including acetate, propionate, lactate, and other organic anions—accumulate in the soil, thereby enhancing carbon sequestration [14]. The paddy mud layer, which is rich in root debris and humus, exhibits a high carbon content in SOM, thereby providing abundant carbon sources for microbial activity. Compared to upland soils, paddy soil SOM exhibits greater stability under waterlogged conditions, which facilitates the formation of soil aggregates and reduces the loss and leaching of inorganic nutrients [17]. In flooded paddy soils, Fe availability represents one of the key factors governing the rate and intensity of SOM mineralization [18]. During rice growth, root oxygen release induces the oxidation of Fe^2^⁺ to Fe^3^⁺ in the rhizosphere, leading to iron plaque formation on root surfaces. This iron plaque interacts with root-exuded organic carbon to form stable Fe-organic complexes, which effectively protect organic carbon from microbial mineralization and enhance carbon sequestration. This process, termed the “iron oxide carbon sink” (or “rust carbon sink”), represents a significant mechanism of rhizosphere carbon storage in paddy systems [19]. Alternating redox conditions induced by anaerobic and periodically flooded-drained cycles in paddy soils significantly influence iron speciation, enhancing its mobility through dynamic Eh fluctuations. In strongly acidic, poorly drained “rusty water” paddy soils, Fe concentrations can reach toxic thresholds of 50–100 mg/kg [20].

### Microbial-mediated mineralization of organic matter in paddy soil

In soil, microbially mediated mineralization and accumulation of organic matter occur simultaneously, with their relative dominance determined by environmental conditions. Under optimal conditions (e.g., adequate moisture, moderate temperature) conducive to microbial growth and metabolism, microbial activity favors mineralization, accelerating organic matter decomposition and nutrient release. Conversely, under suboptimal conditions (e.g., low temperature, water scarcity), microbial metabolism tends to favor humification, resulting in the stabilization of organic matter as humus.

Microbially mediated mineralization plays a key role in nutrient cycling by providing essential nutrients for rice growth. Microorganisms primarily facilitate soil organic matter mineralization through two mechanisms: (i) Intracellular mineralization—microbes metabolize organic compounds within their cells and release the resulting nutrients (e.g., K^+^ and NH_4_^+^) into the soil. For instance, potassium-solubilizing bacteria (KSB) decompose organic potassium compounds, releasing soluble K^+^ [21]. This process enhances K^+^ uptake by rice plants, thereby promoting photosynthetic efficiency and nutrient utilization. (ii) Microbially secreted enzymes accelerate the mineralization process. Recent evidence has demonstrated that zinc fertilizer application enhances the abundance of phosphate-solubilizing bacteria (PSB), the expression of phosphatase-encoding genes, and soil acid phosphatase activity. These changes collectively promote the mineralization of organic phosphorus into plant-available inorganic forms, ultimately improving phosphorus uptake by rice [22]. Furthermore, microorganisms possessing the *phoD* gene (encoding alkaline phosphatase) exhibit an enhanced capacity for phosphorus mineralization. Under phosphorus-deficient conditions, elevated *phoD* gene abundance and increased alkaline phosphatase activity significantly enhance the mineralization of organic phosphorus compounds, thereby increasing plant-available phosphorus pools [23].

Environmental conditions significantly modulate microbial mineralization pathways, thereby influencing the transformation of organic matter in paddy soils. Soil physical structure is a pivotal factor affecting the efficiency of microbial mineralization. For instance, the formation of soil aggregates can limit the direct contact area between microbes and inner organic matter, potentially suppressing the mineralization process to a certain extent [24]. This phenomenon is particularly pronounced in soils amended with biochar, as biochar introduction may alter aggregate stability and consequently affect the availability of substrates to microbes [25]. Furthermore, soil pore structure not only provides a habitat for microbial colonization but also profoundly influences the transport of oxygen, water, and nutrients, all of which are essential for microbial activity. A high soil porosity environment typically favors microbial community growth and metabolism, thus enhancing mineralization [26]. Beyond physical attributes, soil chemical properties also exert considerable influence on the efficiency of organic matter mineralization. For example, temperature, as a crucial environmental factor, significantly impacts the decomposition rate of recalcitrant organic matter. Studies have indicated that an increase in temperature can lead to a higher relative abundance of oligotrophic bacteria in paddy soils, which is correlated with their enhanced activity in promoting organic matter decomposition [27]. Soil pH is another critical chemical parameter that directly affects microbial community structure and the activity of relevant enzymes. This, in turn, influences the overall functional capacity of microbial communities, potentially selectively promoting or inhibiting the mineralization capabilities of specific microbial groups [28]. On the other hand, changes in the soil C/N ratio can also trigger complex microbial responses. A higher C/N ratio may lead to microbial “nitrogen mining” due to nitrogen limitation, consequently accelerating the degradation of native organic matter [29]. However, notably, while improving soil nitrogen availability may promote overall microbial growth, its specific impact on the rate of organic matter mineralization is not uniform and may involve complex threshold effects or response mechanisms [30].

### Microbial-mediated accumulation of organic matter in paddy soil

The accumulation of organic matter is a process in which, under microbial action, soil organic matter undergoes complex biochemical transformations to form humus. Compared to nutrients released directly through mineralization, humus is a stable and vital component of the soil organic matter pool, but it is generally not readily available for crop uptake. Over time, humus can be gradually decomposed by microbes, thereby contributing to soil fertility. Furthermore, humus exhibits high cation exchange capacity, enabling it to effectively adsorb nutrient cations from the soil solution and thus enhance the soil’s nutrient retention capacity.

Microbially mediated organic matter accumulation primarily involves two mechanisms: (i) decomposition of macromolecular organic compounds and (ii) stabilization of soil structure. Through the secretion of extracellular enzymes, microorganisms degrade recalcitrant macromolecules (e.g., cellulose, hemicellulose, and lignin) into low-molecular-weight compounds [31]. A fraction of these decomposition products is assimilated for microbial metabolism, while microbial necromass contributes to the SOM pool. The remaining compounds undergo microbial transformation to form humic substances. Concurrently, microorganisms facilitate soil structure stabilization by promoting aggregate formation and optimizing pore architecture, which physically shields organic matter from rapid decomposition and leaching losses [32].

During rice cultivation, the application of organic fertilizers is equivalent to the artificial input of humus. Through microbial mineralization, these organic fertilizers are decomposed into plant-available nutrients. The predominance of microbially mediated mineralization versus accumulation dynamically shifts in response to (i) fluctuations in nutrient availability and (ii) the alternating flooded and drained conditions characteristic of paddy water management. Flooding phases typically promote accumulation processes by creating anaerobic conditions that inhibit decomposition, whereas drainage periods enhance mineralization through improved oxygen availability.

### Impact of soil microbes on rice root nutrient uptake

Soil microbes serve as a vital bridge between the soil and plants, playing a crucial role in plant nutrient acquisition. The diversity and functional specificity of soil microbial communities can directly or indirectly influence plant root nutrient uptake efficiency, root structure, and resistance to environmental stresses.

#### Pathways of nutrient uptake by rice roots

Rice roots constitute a fibrous root system consisting of seed roots, nodal roots, and lateral roots [33]. As the primary interface for plant–microbe interactions, rice roots develop vigorous lateral roots and root hairs that secrete abundant root exudates, thereby enhancing mineral solubilization and nutrient acquisition for the plant [34].

Root hairs absorb mineral ions through their cell walls and membranes, primarily mediated by transmembrane proteins, including ion channels, transporters, and pumps. These proteins generally exhibit specificity for common nutrient forms, enabling plants to selectively absorb essential ions while excluding toxic ones [35]. For example, in rice, *OsNPF6.5* (also known as *NRT1.1B*) facilitates dual-affinity nitrate transport [36]. Lian et al*.* [37] identified two distinct K⁺ uptake systems in rice: (i) inward-rectifying K^+^ channels, which mediate low-affinity passive transport, and (ii) the KUP/HAK/KT protein family, which is responsible for high-affinity, energy-dependent active transport. Separately, Nozoye et al*.* [38] demonstrated that under Fe deficiency, vesicular localization and transport of *OsNAS2* regulate the synthesis of nicotinamide (NA) and deoxymugineic acid (DMA). These compounds subsequently facilitate Fe chelation by mugineic acid family phytosiderophores (MAs) and promote Fe uptake by root cells.

#### Soil microorganisms promote nutrient uptake by rice root systems

In addition to root-derived chemicals, microorganisms directly enhance nutrient absorption and transformation by (i) promoting plant hormone secretion, (ii) regulating hormone-related gene expression, and (iii) activating ionic channels (e.g., K^+^ channels). Specifically, indoleacetic acid (IAA) promotes root surface area expansion and enhances root elongation and lateral root development. As demonstrated by Khan et al*.* [39], endophytic bacteria regulate rice root growth through bacterial-derived IAA production, which further facilitates nutrient acquisition via increased root surface area. Chandarana, Pramanik, and Amaresan [40] demonstrated that rhizosphere protist activity enhances the activity and survival of IAA-producing plant growth-promoting bacteria (PGPB) in soil. This interaction significantly promotes rice root growth, particularly lateral root development, ultimately modifying root architecture to improve nutrient absorption and transformation. Moreover, Ambreetha et al*.* [41] revealed that PGPR colonization of rice roots modulates the expression of Aux/IAA genes and increases root IAA content, resulting in thicker primary and lateral roots, as well as higher lateral root density. Gibberellins (GA) are key signaling molecules that regulate root elongation. Research indicates that GA promotes root elongation both directly by stimulating cell proliferation and elongation during post-mitotic growth and indirectly by modulating auxin biosynthesis and polar auxin transport[42, 43]. Notably, GA production is not limited to plants; Keswan et al*.* [44] isolated several bacterial strains from soil and rhizosphere environments, including *Pseudomonas aeruginosa*, *Azotobacter* spp., *Thiobacillus* spp., and *Rhizobium* spp., many of which demonstrated the capacity to produce gibberellins.

#### Arbuscular Mycorrhizal Fungi (AMF)-mediated nutrient exchange in rice roots

The symbiotic relationship with arbuscular mycorrhizal fungi (AMF) is a significant strategy for enhancing plant nutrient ion uptake efficiency. Upon infection and colonization, AMF develop an extensive extraradical hyphal system that extends deep into the soil, forming a vast hyphal network. This network greatly expands the nutrient absorption interface of plant roots, thereby significantly increasing plant nutrient uptake and utilization efficiency [45, 46]. The role of AMF is particularly important for rice. In acidic paddy soils, phosphorus readily forms insoluble phosphates with soil metal ions (e.g., aluminum and iron), making it extremely difficult for plants to absorb and significantly reduce phosphorus[47]. Concurrently, nitrification and denitrification processes exacerbate nitrogen volatilization and leaching, reducing the efficiency of organic nitrogen conversion to inorganic forms [48]. AMF, with their unique physiological and biochemical characteristics, can effectively overcome these environmental challenges. Interestingly, the composition of AMF communities symbiotic with rice exhibits high environmental specificity. For instance, in northeastern rice fields, *Glomus* is a common dominant genus in both black soil and saline-alkali soil, with *Paraglomus* showing a higher relative abundance in saline-alkali soil [49].

AMF-mediated nutrient exchange in rice roots primarily occurs through the following mechanisms: (i) Enhanced nutrient acquisition pathways: Colonization by AMF establishes extraradical hyphal networks that translocate carbohydrates synthesized from plant photosynthesis into the rhizosphere, creating a carbon-rich microenvironment that attracts and promotes the colonization and proliferation of other beneficial microbes [50]. This “plant-AMF-bacteria” ternary synergistic complex acts cooperatively, not only strengthening phosphorus activation and transport but also improving the mineralization efficiency of insoluble soil phosphorus through microbial synergistic metabolism, thereby establishing an efficient phosphorus acquisition pathway independent of the plant's traditional root absorption system [51]. Concurrently, the AMF hyphal network can also promote the synergistic uptake and cycling of other nutrients, such as nitrogen and potassium, further enhancing the plant's overall nutrient acquisition capacity. (ii) Maintenance of Root Health and Function: The interaction between AMF and plants is crucial for maintaining host root homeostasis. AMF can enhance systemic resistance by regulating plant signaling pathways (e.g., the jasmonic acid pathway), enabling plants to respond more rapidly and strongly to pathogen infection. AMF can also effectively protect root structure integrity by competing with pathogens for nutrients and binding sites or by secreting antimicrobial metabolites, thereby reducing the risk of pathogen invasion [52]. Additionally, AMF possess a certain compensatory capacity and are able to mitigate losses in root biomass and function caused by pathogen disturbance, thus ensuring sustained plant nutrient uptake [46]. (iii) Regulation of Host Gene Expression: AMF can directly modulate the expression of key plant transporter genes in host plants, thereby optimizing their nutrient uptake and stress responses. For example, AMF can upregulate the expression of the rice phosphorus transporter gene *OsPT11*, enhancing phosphorus acquisition [48]. Further studies have shown that AMF can specifically induce the expression of rice nitrate uptake regulators (e.g., NLP3/PHR2) and their transport complexes in mycorrhizal cells while suppressing their expression in the root epidermis, thereby synergistically improving nitrogen uptake efficiency through the mycorrhizal pathway [53].

### Scale differences in microbial processes in paddy soil

Building upon the systematic elaboration of the mechanisms by which microbial-mediated organic matter mineralization and accumulation promote rice nutrient uptake, previous discussions have primarily focused on rhizosphere microsites, which are strongly influenced by root activity. However, bulk soil, located far from the roots, constitutes the majority of the soil volume. Significant differences in environmental conditions, microbial community composition, and dominant ecological processes exist between these two compartments, profoundly impacting the pathways and efficiency of microbial-driven nutrient transformations [54, 55].

The differentiations between rhizosphere and bulk soil primarily stem from substrate availability and environmental conditions. The well-developed aerenchyma tissue unique to rice roots facilitates oxygen release into specific zones and at particular times, creating a distinctive “alternating oxidizing-reducing” environment in the rhizosphere [56]. This unique environment directly shapes rhizosphere microbial community structure and function. Here, communities are dominated by r-strategist microbes, such as those belonging to *Proteobacteria* and *Bacteroidota*, which drive intense organic matter mineralization. However, the enrichment effects of key microbial functions, such as denitrification, cellulose decomposition, and ureolysis, are relatively attenuated in the rhizosphere [57]. In contrast, bulk rice soil is characterized by a stable anaerobic state over the long term, hosting microbial communities predominantly composed of oligotrophic, K-strategist microbes adapted to slow turnover and strict anaerobes, for instance, members of *Chloroflexi*, *Acidobacteria*, and certain *Proteobacteria* [58]. Due to limited fresh organic carbon input, microbes in this zone are better adapted to low-nutrient and hypoxic environments, exhibiting lower metabolic activity and turnover rates, which leads to significantly reduced organic matter mineralization. Organic matter decomposition relies more heavily on fermentation and anaerobic respiration pathways, utilizing electron acceptors such as iron and manganese [59].

Notably, rice soils exhibit a lack of pronounced enrichment for specific functions common in upland soils. This may be because organic compounds exuded by rice roots under flooded conditions are easily diluted and rapidly dispersed in the water column, resulting in less pronounced local concentration gradients compared to upland soils [60]. Consequently, the strong directional selection effects on specific functional microbial groups are weakened, leading to a more homogeneous distribution of microbes in rice soils. This difference in scale suggests that strategies effective in upland crop systems for enhancing nutrient cycling by manipulating rhizosphere microbes may not be directly applicable to paddy fields. Future research needs to develop microbial management theories and technological measures tailored to the unique anaerobic-amphibolic interfaces characteristic of rice soils.

## Effect of fertilizer application on the agricultural system of paddy fields

Fertilizers (including inorganic and organic forms) represent a primary nutrient input for intensive rice production systems. Their application has a strong influence on soil fertility and shapes the microbial community composition in rice paddies.

### Effect of fertilizer application on soil fertility in paddy

The main factors determining soil fertility include organic matter content, plant-available inorganic nutrients, and soil physical properties. SOM serves as a fundamental indicator of soil fertility [61]. SOC, the carbon-containing portion of SOM, represents its most important component.

**Fig. 2:**
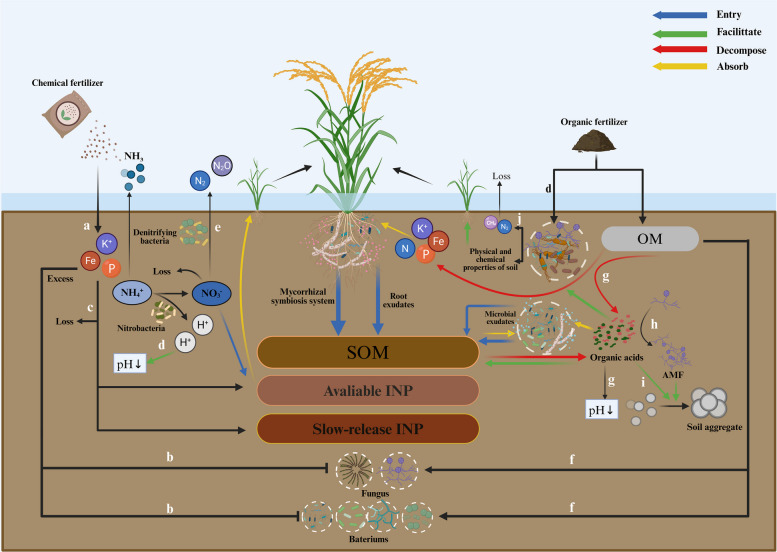
Effect of fertilizer application on paddy soils and microorganisms. The application of inorganic fertilizers significantly increases the readily available inorganic nutrients in soil (e.g., N, P, K) in the short term (**a**), leading to alterations in soil physicochemical properties. These changes directly influence the soil microbial community, resulting in shifts in microbial composition and abundance; for example, excessive P fertilization suppresses the abundance of AMF and *Actinobacteria* in the soil (**b**). Flooded paddies promote the loss of certain readily available inorganic nutrients (such as Fe^3+^) through runoff and leaching or their sequestration into inert INP (**c**). NH_4_^+^ is oxidized via nitrification, producing H^+^, which is a primary contributor to soil acidification and pH decline (**d**). The anaerobic environment in flooded soils facilitates denitrification processes, gradually reducing NO_3_^−^ to gaseous forms such as N_2_ and N_2_O (**e**). Organic fertilization markedly increases soil organic matter content and promotes the proliferation of specific microbial groups, such as methanogens and denitrifiers (**f**). Long-term application of organic amendments can lead to the accumulation of soil organic carbon, forming a stable carbon pool. The anoxic conditions induced by flooded rice cultivation suppress the activity of most microbes, resulting in incomplete organic matter decomposition and the production of organic acids, which lower soil pH (**g**) and affect microbial communities, such as changes in AMF populations (**h**). Organic acids, containing organic carbon, can effectively facilitate soil aggregate formation (**i**). The decomposition of organic matter consumes large amounts of oxygen, exacerbating hypoxic conditions in rice soils. This deepened anaerobic environment creates favorable conditions for methanogens, which utilize small molecular organic compounds for metabolism, releasing CH_4_ (j). OM: organic matter; SOM: soil organic matter; AMF: arbuscular mycorrhizal fungi; INP: inorganic nutrient pool

Organic fertilizers are materials derived from the fermentation and maturation of animal and plant residues, excreta, and other organic wastes. While organic fertilizers exhibit considerable diversity in type, encompassing categories such as composts, green manures, and oilseed cakes, their primary constituents can be broadly defined as organic matter, essential nutrients, humic substances (including fulvic acid and humic acid), and biologically derived structural components. Of these, organic matter represents the foundational component of organic fertilizers, predominantly sourced from components such as starch and proteins of both animal and plant origins. Organic fertilizers primarily regulate soil fertility by directly increasing the SOM content of rice soils, maintaining or improving SOM stability, and enhancing soil structure and physicochemical properties [62]. Studies have demonstrated that the substantial organic matter content in organic fertilizers can directly increase the SOM content of rice soils [63]. Furthermore, organic fertilizers can enhance SOM stability and increase SOM content by increasing the concentration of dissolved organic matter (DOM), the readily bioavailable fraction of SOM [64]. Feng et al*.* [65] found that organic fertilizers improved SOM stability in soil by increasing the aromaticity of water-soluble substances and promoting the formation of recalcitrant organic compounds, such as fulvic acid (FA) and humic acid (HA), within the SOM pool. Organic fertilizers can directly enhance the ability of rice to fix nitrogen, photosynthesize, and absorb minerals and can also help crops recruit specific beneficial microbial communities to improve the physicochemical properties of the soil, such as soil enzyme activity, soil pH, and soil particle porosity, which can indirectly promote the growth of rice [66]. Moreover, after the application of organic fertilizers, the organic matter is easily decomposed by microorganisms to produce organic acids, leading to a decrease in soil pH (Fig. 2). This finding can alleviate the adverse effects of high pH in highly alkaline soils and create an environment suitable for rice growth and nutrient uptake in saline soils[66]. Furthermore, high-quality plant litter promotes the formation of stabilized organic carbon fractions (e.g., MAOM). However, when these stabilized fractions reach saturation capacity, litter quality ceases to enhance soil carbon stocks [67].

Inorganic fertilizers are synthesized or mineral-derived compounds that exist primarily as inorganic salts. Their principal components are single or complex mineral nutrients (Fig. 2). The major categories of inorganic fertilizers include nitrogen (N) fertilizers, phosphorus (P) fertilizers, and potassium (K) fertilizers. Nitrogen fertilizers are commonly formulated as urea or ammonium nitrate; potassium fertilizers are formulated as potassium chloride or potassium nitrate; and phosphorus fertilizers are formulated as superphosphate or triple superphosphate. Inorganic fertilizers alter the quality of plant litter after plant uptake, effectively supplementing the soil’s fast-acting inorganic nutrient pool. This, in turn, increases the likelihood of direct microbial contact and decomposition of plant litter, accelerating the conversion of plant litter to stable SOM, enhancing soil fertility, and promoting crop growth. Zhou et al*.* [68] reported that long-term delayed application of chemical fertilizers, compared to normal application, increased the root dry weight of rice plants at the spike differentiation stage. This not only increased the input of root residues into the soil later but also improved SOM by stimulating lignin degradation and promoting the accumulation of microbial necromass. Inorganic fertilizers stimulate microbial enzyme production, which enhances plant litter decomposition and nutrient bioavailability, ultimately promoting SOC accumulation in agricultural systems [69]. However, extensive research has demonstrated that prolonged excessive fertilizer application causes (i) soil fertility decline, (ii) environmental contamination (e.g., groundwater pollution), and (iii) nutrient imbalances. These effects collectively impair rice nutrient acquisition and reduce crop yields [70]. Many commonly used chemical fertilizers tend to be neutral or weakly acidic. Following nutrient uptake, plants release CO_2_ through respiration. CO_2_ combines with soil water to form carbonic acid (H_2_CO_3_), which dissociates into hydrogen ions (H^+^), thereby contributing to soil acidification and negatively impacting crop yield (Fig. 2).

Some studies have documented that long-term fertilization can improve soil fertility. However, other research has reported that the prolonged application of inorganic fertilizers leads to severe degradation of red soil, characterized by high acidity, low nutrient content, and disturbed, unbalanced ecosystems [71]. These conflicting results between studies are closely related to the specific soil types investigated. Based on soil properties, Chinese paddy soils can be broadly classified into mature paddy soils (characterized by slightly acidic to neutral pH), acidic paddy soils, and saline-alkalized paddy soils. In southern China, long-term cultivation of double-cropping rice fields typically results in the development of mature paddy soils with stable nutrient levels and abundant organic matter. Studies have demonstrated that the combined application of chemical fertilizers with organic fertilizers or straw incorporation yields superior productivity and environmental benefits compared to the exclusive use of chemical fertilizers [72]. Notably, the organic fertilizer + chemical fertilizer combination proved more advantageous than the other two methods. Practical production insights suggest that an optimal substitution ratio for organic fertilizers should not exceed 50% [73]. Currently, a ratio of 30% organic fertilizer to 70% chemical fertilizer is commonly adopted in southern double-cropping rice fields [72, 74]. In acidic paddy soils, a combination of 70% organic fertilizer and 30% chemical fertilizer has been shown to more effectively enhance soil organic carbon sequestration rates, nitrogen use efficiency, and crop yields [75]. Under conditions of elevated temperature and high nitrogen fertilization, prevalent practices of straw return and manure application in current double-cropping rice systems markedly enhance emissions of methane (CH_4_) and nitrous oxide (N_2_O). In contrast, the combined application of fertilizer and biochar demonstrates the potential to substantially mitigate the total warming potential stemming from these three major greenhouse gases while concurrently sustaining or even improving crop yields [76]. For acidic soils exhibiting inhibited nitrification and weak nutrient retention, it is advisable to apply ammonium nitrogen in split doses and implement pH improvement strategies [77]. Poultry manure, especially chicken manure, can be quickly converted for rice utilization due to its rapid nitrogen mineralization rate, significantly increasing crop yield in the short term. When co-applied with chemical fertilizers, it maximizes yield and significantly improves soil properties [78]. The performance of pig manure lies between that of chicken and cattle manure; it provides considerable yield increases and is particularly effective in reducing nitrogen loss from surface water, decreasing ammoniacal nitrogen by 21.55% and nitrate nitrogen by 50.60% [79]. In saline-alkalized paddy soils, the soil condition of saline-alkali paddy soils is predominantly characterized by high salinity, elevated pH (alkalinity), and poor structural integrity. On the one hand, these conditions can temporarily sequester certain heavy metals at high pH levels via chemical precipitation and adsorption, thereby diminishing their immediate bioavailability. On the other hand, high salt content, particularly elevated chloride ion concentrations, can promote the formation of soluble metal complexes, facilitating heavy metal migration. Concurrently, the compromised soil structure impedes the effective leaching and dilution of heavy metals, thus exacerbating the risk of their long-term accumulation in the soil. However, the influence of fertilization on heavy metal-contaminated saline-alkali paddy soils represents a comparatively under-researched aspect within current scientific investigations. Multiple studies have highlighted that heavy metal elements carried by organic fertilizers are challenging to eradicate during the fermentation process. Consequently, there exists a potential risk of metal accumulation when applying organic fertilizers to rice soils [80–82]. Low levels of organic fertilizers + chemical fertilizers can increase stable organic compounds and reactive minerals, such as that achieved through the mixed application of 70% inorganic fertilizers and 30% swine manure, thereby promoting the formation of organic-mineral complexes within soil colloids. This approach represents a more effective fertilization strategy for enhancing the storage of organic chemicals and compounds in saline-alkalized rice soils [83, 84]. Furthermore, the use of treated domestic wastewater in lieu of sludge compost has also shown high potential: compared to applying only mineral fertilizers, it not only increased rice yield by 27% and protein content by 25% but also significantly reduced the accumulation risk of heavy metals in rice grains and soil [85]. Although traditional organic fertilizer application may lead to unavoidable heavy metal contamination effects, its positive benefits may, in many cases, outweigh its negative impacts.

### Effect of fertilizer application on paddy soil microorganisms

The effects of fertilizer application on soil microorganisms can be broadly classified into direct effects on microbial community composition, abundance, and activity and indirect effects by altering soil physicochemical properties, such as pH [86]. Notably, fungal and bacterial communities exhibit distinct response thresholds to various fertilizer types.

#### Effect of fertilization on paddy soil fungal communities

AMF are often the main focus of research on soil-colonizing fungi. These fungi also include members from the *Ascomycota* spp., *Basidiomycota* spp., and *Rozellomycetes phylum* spp. Fertilizer application can influence the fungal community in rice soils through both direct and indirect mechanisms.

The direct pathway is reflected in fertilization effects on fungal community composition, diversity, and abundance. Excessive inorganic fertilizer application disrupts soil nutrient ratios, inhibiting the growth of fungi reliant on specific nutrient balances (Fig. 2). This alteration of fungal ecological niches ultimately leads to a reduction in community diversity and abundance. For example, excessive synthetic nitrogen fertilization inhibits AMF development, leading to reduced AMF abundance (including that of external mycelium), decreased soil fungal diversity, and altered community composition. In contrast, some literature has demonstrated that moderate mineral nitrogen fertilization, relative to unfertilized conditions, decreases fungal abundance by 44% but shows little effect on crop yield and fungal diversity [87]. Differences in experimental outcomes, potentially attributable to the timing of fertilizer application, could explain these observations. Moreover, numerous studies have found that prolonged mineral fertilization inhibits the functioning of fungi in nutrient cycling by reducing their abundance. These changes ultimately impair plant uptake of essential nutrients such as phosphorus [88]. Long-term application of inorganic phosphorus fertilizer increases soil P saturation levels while reducing AMF colonization in root systems [89]. However, Ye et al*.* [90] found that long-term mineral fertilization had little effect on fungal community composition, which may be due to the small difference in SOC content between the control soil and the inorganic fertilizer soil, which cannot drive changes in fungal communities. Several studies have demonstrated a positive correlation between fungal abundance and SOM content [91]. Organic fertilizers enhance soil available nutrient content and carbon/nitrogen storage, thereby supplying substrates for fungal growth and promoting fungal community diversity and abundance [88, 92]. However, excessive organic fertilizer application can lead to soil C:N ratio imbalance, elevated nutrient availability, and reduced plant dependence on mycorrhizal networks [93, 94]. Therefore, judicious organic fertilizer application is essential to maintain soil–plant-microbe balance while ensuring high crop productivity.

The indirect pathway involves fertilizer-induced alterations in the soil physicochemical properties, which subsequently influence fungal community structure and abundance [95]. Specifically, in black soils, the combined application of NPK fertilizers with manure has been demonstrated to enhance fungal community diversity and restore fungal communities toward their natural state. In contrast, the exclusive use of inorganic fertilizers decreased soil pH and led to a reduction in beneficial fungal populations [95] (Fig. 2). The primary drivers are soil accumulation of excess NH_4_^+^ and available phosphorus (AP). NH_4_^+^ excess induces soil acidification and alters bacterial communities. Conversely, AP accumulation significantly diminished fungal diversity in both the rhizosphere and endosphere. Xylaria fungi are particularly affected; these fungi are known to regulate the expression of plant nutrient transporter genes, including the iron-regulated *ysl15* and the nitrate transporter *NRT1.1A*. These fungi play crucial roles in soil nutrient cycling and contribute significantly to plant resilience against [96, 97]. In contrast, the combined application of organic and inorganic fertilizers in paddy soils improves physicochemical properties and enriches two key fungal phyla – *Ascomycetes* spp. and *Basidiomycetes* spp. These fungi mediate soil N/C cycling through cellulose degradation (via *cbhI* gene-encoded enzymes) and contribute to SOC maintenance [88, 98]. Studies have demonstrated that the co-application of organic and inorganic fertilizers modulates soil pH and substrate stoichiometry (C:N ratios), thereby creating favorable ecological niches for cellulose degradation. This environment selectively enriches specific fungal taxa harboring the functional gene *cbhI*. These fungi constitute critical microbial guilds responsible for the decomposition of recalcitrant organic carbon, and their abundance is intrinsically linked to soil carbon cycling dynamics [99]. Soil structure, a critical determinant of soil health, governs the storage and transport of water, gases, and nutrients, consequently regulating fungal community abundance and activity. As SOC serves as the primary binding agent for aggregate formation, fertilizer applications (both organic and inorganic) can directly or indirectly enhance aggregate stability (Fig. 2). However, excessive fertilizer application introduces ionic overload, leading to the dispersion of soil colloids and particles, thereby disrupting aggregate formation in paddy soils [100]. This deterioration of soil structure disrupts nutrient and water distribution patterns, leading to a significant decline in fungal community abundance.

#### Effect of fertilizer application on paddy soil bacterial communities

In contrast to fungal communities, bacterial communities respond differently to fertilization due to their distinct nutrient requirements and ecological niches. In the short term, both inorganic and organic fertilizer applications stimulate shifts in bacterial community composition and abundance. However, long-term organic fertilization demonstrates more substantial positive effects, particularly enriching Bacteroidetes, Firmicutes, and Actinobacteria. Agronomic practices such as periodic flooding/drainage establish unique microbial communities in paddy soils that diverge significantly from dryland systems (Fig. 2). For instance, soil anoxia leads to the enrichment of anaerobic microorganisms (e.g., methanogens and denitrifiers) in paddy soil. Organic fertilizer application helps to maintain a dynamic equilibrium in the enrichment of various characteristic microorganisms in rice soils over time.

Short-term moderate fertilizer application enhances bacterial abundance; however, prolonged use leads to element accumulation, which adversely affects soil health (e.g., acidification caused by pH reduction). In paddy systems, urea/ammonia fertilization induces nitrification to release H^+^ ions, resulting in soil acidification and leading to nitrogen loss due to the mobility of nitrate [101]. These changes ultimately result in a decline in the abundance of key C/N-cycling bacteria, such as Bacteroidota and diazotrophs, in paddy soils. It has been found that high levels of phosphorus after the application of inorganic P fertilizers inhibit the growth of bacteria carrying the *phoD* gene, which encodes enzymes capable of mineralizing organic phosphorus into inorganic forms. The study shows that the relative abundance of *Actinobacteria* spp. and *Cyanobacteria* spp. in unfertilized soils is generally higher than that in phosphorus-fertilized soils [23]. This occurs because phosphorus fertilization increases soil phosphorus availability, thereby alleviating the need for microbes and plants to mineralize organic phosphorus or solubilize inorganic phosphorus. Through a negative feedback mechanism, this reduced demand suppresses the abundance of functional genes such as *phoD*, consequently diminishing the populations of microorganisms capable of organic phosphorus mineralization [102].

Moderate organic fertilizer inputs enhance bacterial community abundance and diversity in paddy systems. Sun et al*.* [103] demonstrated that 10% manure application optimizes plant growth while preserving high bacterial diversity. However, excessive manure application, despite increasing soil total nitrogen (TN) and SOM content, may elevate heavy metal concentrations, suppress soil enzyme activity, and alter bacterial community structure. Notably, combined rice straw and organic fertilizer application fosters more favorable conditions than conventional chemical fertilizers for enriching ureolytic (*ureC*-associated) and chitinolytic (*chiA*-associated) bacterial communities in double-cropping rice rhizosphere soils [104]. Dong et al*.* [105] further demonstrated that organic fertilizer application more strongly influences *Actinobacteria* spp. in paddy soils than inorganic amendments. These fertilizers provide diverse substrates for microbial growth and exogenous beneficial microorganisms that collectively enhance soil bacterial biomass. During the process of organic fertilizer input, pH had a large impact on the composition and abundance changes of soil bacterial communities. Although the mechanism by which biochar application affects bacterial community composition is not completely understood, Ali et al*.* [106] found an increase in pH and an increase in SOM content in the soil by pairing biochar with inorganic nitrogen fertilizer. The abundance of most acidophilic bacteria in the soil gradually decreased, and the abundance of *Bacteroides* spp. (containing abundant genes encoding GH glycoside hydrolases) and *Actinobacteria* spp. (containing *chi* genes, chitosanase genes *csnA*, etc.) increased. These bacterial groups utilize biochar as a carbon source and contribute to soil carbon and nitrogen cycling through the decomposition of cellulose and chitin. These microbial shifts ultimately enhance rice growth and biomass accumulation by improving nutrient availability during reproductive stages [107, 108]. However, some studies have also observed no significant difference in soil bacterial alpha diversity between fertilized and unfertilized groups. This observation could be attributed to factors such as the alternating flood-and-dry conditions in the rice fields and the limited sampling spatial distance, which led to minimal variations in soil pH. Consequently, this resulted in a very subtle difference in soil bacterial alpha diversity [109]. Furthermore, while short-term organic fertilization enriches methanogens in paddy soils, prolonged application significantly increases Methylobacterium (methane-oxidizing bacteria) and Azotobacter (nitrogen-fixing bacteria) [86] (Fig. 2). This functional shift may mitigate methane emissions while enhancing atmospheric nitrogen fixation. Methanogenic archaea and methane-oxidizing bacteria are both enriched in paddy soils due to paddy soil’s unique physicochemical properties. Methanogens predominantly inhabit the deeper, anaerobic soil layers where oxygen is scarce, whereas methane-oxidizing bacteria colonize the surface and micro-oxic zones. The latter utilize methane derived from methanogenic activity as their carbon and energy source, supported by oxygen diffusion from rice roots and oxic microniches in the rhizosphere. Additionally, microbial iron reduction converts Fe^3+^ to Fe^2+^, forming complexes that further stabilize the micro-oxic conditions in the surface soil [110].

Different crops, soil environments, and fertilization practices, and even at different stages of plant growth and development, can affect microbial abundance and activity [111]. In real paddy field systems, multiple environmental factors interact and profoundly shape the structure and function of microbial communities. Following fertilizer application, soil bacterial communities exhibit greater sensitivity to field-scale heterogeneity than fungal communities. Topography is a crucial dimension influencing soil nitrogen budgets, with its effects varying across scales. At the macro-scale, significant topographic relief considerably impacts nitrogen distribution and utilization. For instance, after organic fertilizer application, valley bottom areas exhibit superior nitrogen uptake, biomass, and yield compared to slopes. This is attributed to higher abundances of denitrification and bacterial ammonia assimilation genes in low-lying areas, indicating enhanced nitrogen retention and effective supply capacity [112]. However, micro-scale topographic management yields different outcomes. Under a high-elevation context, ridge cultivation retains more residual soil nitrogen and reduces water-related nitrogen losses by approximately 30% compared to conventional flat cultivation and flooded paddies [113]. Soil pH also plays a critical regulatory role. pH is positively correlated with the abundance of ammonia-oxidizing archaea (AOA) and ammonia-oxidizing bacteria (AOB), a ratio that is key to regulating the conversion of NH_4_^+^ to NO_3_^⁻^. Therefore, variations in pH directly influence the ratio of ammonium to nitrate nitrogen in the soil [114]. Furthermore, altered cultivation practices exert profound influences on the diversity and composition of soil bacterial communities. For example, shallow tillage leads to a 10.5% decrease in the relative abundance of *Nitrospirae*, potentially exacerbating nitrous oxide production and loss, thus being detrimental to rice yield. Conversely, deep tillage promotes the high interconnectedness of bacterial networks, with significantly enhanced inter-community competition, as evidenced by a 1.64-fold increase in the proportion of negative correlations between bacterial taxa [115]. In summary, considering field-scale spatial heterogeneity is not only a prerequisite for accurately predicting the ecological effects of fertilization but also a crucial basis for developing site-specific management strategies and optimizing resource utilization.

### Effects of fertilization on the trade-off between soil carbon sequestration and greenhouse gas emissions

Greenhouse gas (GHG) emissions are a major driver of global climate change, with methane (CH_4_) and nitrous oxide (N_2_O) being the two primary agricultural greenhouse gases [116]. In agricultural production, the selection and application of fertilizers directly influence the formation and release of greenhouse gases in the soil.

It is generally believed that fertilization increases SOC while also increasing CH_4_ and N_2_O emissions from rice paddies, with the application of organic fertilizers exacerbating this effect. Studies have found that during rice cultivation, the use of straw or manure not only increases rice yields but also selectively promotes the growth of specific methanogenic populations by providing a carbon source, thereby significantly increasing CH_4_ emissions [117, 118]. However, some studies have found no significant differences in CH_4_ emissions among fertilization treatments under optimized fertilization practices, challenging the notion that “organic fertilizers significantly increase CH_4_ emissions” [119]. For example, while the abundance of the *mcrABC* gene is typically positively correlated with CH_4_, Wang et al*.* found that this relationship does not exist. There are two possible reasons for this: first, the complex organic carbon sources in organic fertilizers are preferentially utilized by heterotrophic microorganisms, thereby reducing the substrates essential for methanogenic bacteria (such as H_2_, CO_2_, and acetate) and inhibiting their growth and reproduction. Second, rice cultivation may influence the abundance of methane metabolism genes as well as the processes of CH_4_ oxidation and production [120]. Under compost application during rice cultivation, CH_4_ emissions were reduced by 20%, and the SOC content increased annually, whereas SOC losses occurred under fresh manure application. Overall, the results indicate that composting is a sustainable management strategy that effectively reduces greenhouse gas emissions and promotes soil carbon sequestration while ensuring rice yields [121]. In addition, recent studies have increasingly demonstrated that biochar—a product obtained by pyrolyzing biomass under high-temperature, oxygen-deprived, or anaerobic conditions—plays a significant role in promoting soil carbon sequestration and mitigating greenhouse gas emissions. The application of biochar enhances SOC accumulation by increasing the organic matter content in soil aggregates and promoting its complexation with iron, aluminum, and manganese [122]. When applied in combination with rice straw, it not only provides a continuous and stable input of carbon, leading to the most robust and stable growth in SOC sequestration but also effectively suppresses CH_4_ emissions [118].

Inorganic fertilizers, such as nitrogen fertilizers, help expand leaf area and promote the transport of carbon produced by photosynthesis to the roots, thereby increasing root exudates from methanogenic bacteria. However, their impact on SOC accumulation weakens after reaching a certain threshold; thereafter, excessive N inputs increase greenhouse gas emissions and lead to eutrophication of aquatic systems [123, 124]. A growing body of research indicates that the combined use of inorganic fertilizers, organic fertilizers, and soil conditioners can effectively control CH_4_ and N_2_O emissions while increasing SOC. Wang et al*.* found that the application of organic–inorganic compound fertilizers effectively increased the relative abundance of nitrifying bacteria (*Bacteroides* spp.) and nitrogen-fixing bacteria, thereby controlling N_2_O emissions in saline-alkali paddy ecosystems [125]. Galgo et al*.* found that a silicate modifier derived from iron slag not only suppressed CH_4_ and N_2_O emissions but also increased SOC accumulation, contributing to long-term carbon sequestration [126]. Yun et al*.* found that treatments combining silicates and compost significantly reduced methane (CH_4_) and nitrous oxide (N_2_O) emissions by neutralizing soil pH and regulating denitrification processes [127]. These findings provide strong evidence that silicates can serve as a sustainable strategy to reduce greenhouse gas emissions while maintaining high crop productivity.

Although biochar, soil conditioners, and other agents play a crucial role in stabilizing soil carbon sequestration and controlling greenhouse gas emissions, we must recognize that their one-time, large-scale application can lead to adverse consequences over time.

## Main mechanisms and influencing factors of microbial mediation in carbon storage of paddy soils

SOC, as an important component of soil organic matter, plays a crucial role in soil fertility and nutrient absorption by plants due to its storage capacity in the soil. Soil carbon stock (SCS) represents the total carbon mass stored within a defined soil volume (area × depth). As a key indicator of both soil carbon sequestration potential and ecosystem carbon budgets, the SCS comprises two principal forms—organic carbon and inorganic carbon—that collectively regulate soil fertility and climate feedback mechanisms.

### Paddy soil carbon pool

**Fig. 3:**
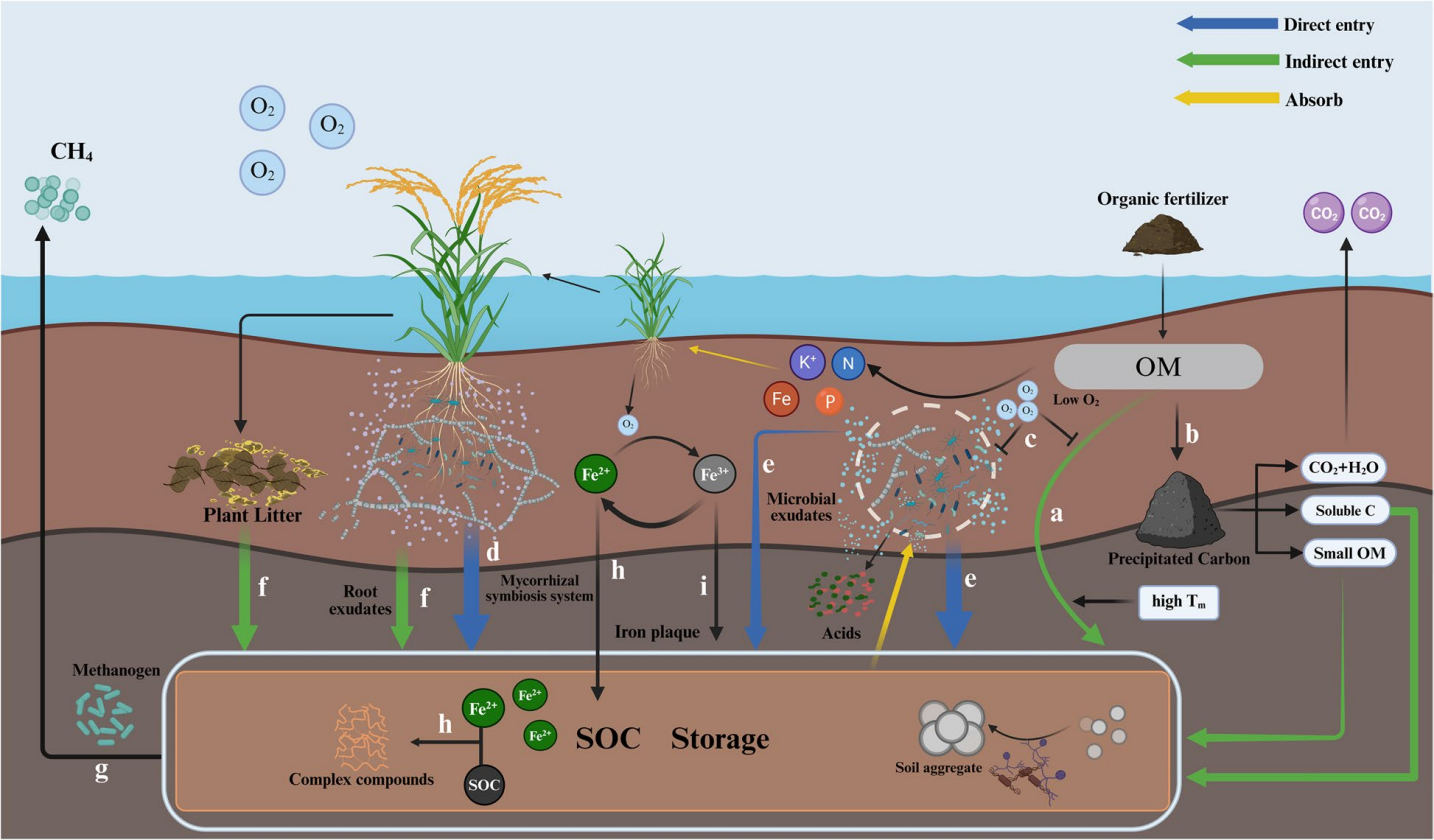
Main mechanisms of microbial mediation in carbon storage of paddy soil. A portion of the applied organic amendments is decomposed by the microbial community, directly increasing SOC storage in rice paddies (**a**), while another fraction is transformed into recalcitrant carbon that is retained in the soil (**b**), subsequently undergoing slow microbial decomposition. The unique low-oxygen environment in rice soils inhibits microbial activity, slowing organic matter turnover and, consequently, limiting the rate at which carbon from organic amendments is directly incorporated into SOC storage (**c**). Fungal communities in rice soils can form mycorrhizal symbioses with rice roots. The subsequent death of these symbiotic associations directly contributes to SOC storage (**d**). Furthermore, microbial metabolites and cell debris represent significant direct sources of SOC (**e**). Microorganisms indirectly increase SOC input by promoting nutrient uptake by rice roots, enhancing plant biomass, and stimulating the release of carbon-containing root exudates (**f**). Flooded conditions in rice paddies accelerate the accumulation of carbon-containing root exudates within the soil. Under anaerobic conditions, methanogens become enriched, consuming SOC and producing methane (**g**). Moreover, the anaerobic reduction of Fe^3^⁺ to Fe^2^⁺ enhances the binding capacity of mineral surfaces for SOC, forming complexes (**h**). In contrast, the release of small amounts of oxygen from rice roots promotes the oxidation of some Fe^2^⁺ back to Fe^3^⁺, resulting in the formation of insoluble iron plaques (**i**), which protect SOC storage through a physical barrier. OM: organic matter; SOC: soil organic carbon; T_m_: temperature

The main components of soil carbon pools in rice paddies are organic carbon, inorganic carbon, and organic–inorganic composites. The organic carbon fraction contains both relatively labile and relatively recalcitrant components. The stable components of the inorganic carbon fraction are primarily carbonate compounds. Organic–inorganic complexes consist mainly of organic carbon complexes with clay minerals/metal oxides [128]. These differences in composition result in significant distinctions between the carbon pools in paddy soils and upland soils in terms of carbon sequestration capacity and microbial-mediated mechanisms. Compared with upland soils, paddy soils exhibit higher organic carbon content and greater carbon sequestration capacity [129]. In terms of carbon pool composition, paddy soils have significantly higher levels of particulate organic carbon and mineral-associated organic carbon than upland soils [130]. Moreover, the distinctive flooded cultivation system in paddy soils alters microbial-driven nutrient cycling, leading to divergent SOM turnover dynamics compared to upland soils [131]. Taking iron as an example, iron (oxyhydr)oxides sorb relatively small and soluble organic molecules during the alternating wetting and drying cycles of paddy soils, promoting catalytic oxidation and polymerization and thereby accelerating the formation of larger organic molecules. After the reduction and dissolution of iron, these molecules bind to clay minerals and are incorporated into mineral-associated organic carbon, thus enhancing carbon sequestration in paddy soils [132]. In paddy soils under submerged conditions, Fe^3+^ in iron (oxyhydr)oxides is reduced to Fe^2+^, facilitating the formation of mineral-organic complexes. This process inhibits microbial decomposition, thus stabilizing the carbon pool and extending SOC turnover time by 2–threefold compared to upland soils [133]. During rice root growth, limited oxygen release promotes the oxidation of Fe^2+^ to Fe^3+^, resulting in iron film formation. These films adsorb organic matter, forming thermodynamically stable complexes that act as physical barriers to protect SOC and reduce microbial access [19] (Fig. 3). The mineralization and accumulation of SOC are dynamic processes. During this process, microbial carbon use efficiency (CUE), defined as the efficiency with which microorganisms convert absorbed carbon into their own biomass carbon, plays a crucial role as a key parameter characterizing the formation rate of SOM. Studies have shown that CUE is not only a core determinant of SOC stocks but also directly influences how microbial decomposers regulate the carbon balance between the atmosphere and the soil [134, 135]. Building on previous research, Yang et al. proposed that microbial CUE can also indirectly affect particulate organic carbon (POC) and MAOC through pathways involving microbial biomass and necromass, a mechanism termed the “continuous burial effect” [136]. When microorganisms allocate more carbon to their own growth (high CUE), less energy is lost through respiration, resulting in reduced CO_2_ release from organic matter decomposition. Consequently, microbial mineralization of organic matter decreases; conversely, low CUE accelerates mineralization. Simultaneously, the microbial metabolites and necromass produced under high CUE can enhance SOC sequestration [137, 138].

It is noteworthy that microbial CUE is not fixed but is regulated by both abiotic and biotic factors. Among abiotic factors, climate change affects soil temperature and water availability, which directly impact microbial metabolism. Generally, under high-temperature conditions, microbial respiration exceeds growth, so CUE typically decreases with rising temperature [139]. Furthermore, soil pH is a major factor influencing CUE. Studies have found that under future climate change scenarios, the increase in CUE is greater in alkaline soils than in acidic soils. This is because microorganisms in low-pH conditions require more energy to overcome H^+^/Al^3+^ stress. In low-pH environments, resource competition between bacteria and eukaryotes intensifies, and these changes ultimately lead to a reduction in CUE [140]. The structure and diversity of the microbial community also significantly affect CUE. Microbial taxa are often classified into r-strategists and K-strategists based on their growth, reproduction, competition, and adaptation strategies. Among these, r-strategists (copiotrophs, typically dominated by bacteria) are characterized by rapid growth and dominance in environments rich in labile organic carbon, often accompanied by faster net carbon mineralization rates. In contrast, K-strategists (oligotrophs, typically dominated by fungi) grow slowly but exhibit higher efficiency in utilizing recalcitrant organic carbon, allowing them to survive in resource-limited environments [141]. Interestingly, some studies have found that the enrichment of oligotrophic bacteria can actually increase microbial CUE because these bacteria mineralize less native SOC to meet the same carbon demand [142]. Current research findings on the specific effects of different microbial taxa on CUE are not entirely consistent. For example, Margarida Soares's study indicated that CUE is highest when the fungal-to-bacterial ratio (F:B) is low [134]. In contrast, Luiz A. Domeignoz-Horta and colleagues found that temperature and humidity can indirectly influence CUE by regulating the structure and diversity of the microbial community and that in their study model, the presence of fungi had a positive effect on CUE [143]. These seemingly contradictory conclusions suggest that a systematic and accurate understanding of how different microbial taxa determine CUE levels is still lacking, and further analysis incorporating specific environmental factors is needed.

### Main mechanisms of microbial-mediated carbon storage in paddy soils

In paddy soils, microorganisms play a crucial role in SOC storage, mediating the decomposition of organic matter into bioavailable nutrients for plant uptake [144]. As key drivers of biogeochemical cycles, their influence on SOC storage operates through two primary mechanisms: direct and indirect pathways.

#### Direct mechanism

Direct contributions of microorganisms to soil carbon stocks primarily occur through two mechanisms: (i) plant-root symbiosis, where microbes receive plant-derived organic carbon and facilitate its transfer into soil; and (ii) microbial biomass and necromass accumulation. Necromass comprises both extracellular metabolic byproducts released during microbial activity and cellular residues after death (Fig. 3). The carbon content of these pools is regulated by CUE [135].

Among the common fungal groups in agricultural systems, AMF exert the most significant influence on soil carbon stocks [145]. As obligate biotrophs, AMF form mutualistic associations with a wide range of host plants. The symbiotic relationship involves the plant allocation of photosynthate-derived C to AMF through root exudates. Following C assimilation by AMF, a portion of this organic C is translocated to deeper soil layers, where it is physically protected from microbial decomposition. This process enhances soil C cycling and contributes to the stabilization of SOC pools [146]. In rice paddies, root exudation provides substantial C inputs to the soil system while simultaneously serving as a key C source for anaerobic microbial communities (e.g., methanogens and sulfate-reducing bacteria). The flooded conditions characteristic of paddy soils further promote the accumulation of root-derived C in soil organic matter [147]. The composition of soil microbial communities, particularly the fungal/bacterial ratio, significantly influences SOC pools. Soils with a higher fungal dominance typically exhibit greater carbon storage capacity [148]. Among fungi, AMF are keystone players in agricultural systems due to their extensive hyphal networks with plant roots. Microorganisms with higher CUE demonstrate superior carbon assimilation efficiency, enabling them to allocate more decomposed SOC into biomass rather than respiration. This metabolic strategy effectively reduces C losses and enhances long-term SOC sequestration [135]. Compared to upland crops, rice plants exhibit higher root carbon input and rhizodeposition while exhibiting lower oxygen demand. These conditions promote slower microbial turnover and enhanced carbon assimilation. Research demonstrates that oxygen-limited conditions in paddy soils support microbial biomass approximately twofold higher than in upland systems, owing to suppressed microbial activity and decelerated turnover kinetics [133]. Crucially, Chen et al*.* [149] established that organic amendments (e.g., straw, manure) stimulate microbial growth and necromass production, ultimately elevating SOC content.

#### Indirect mechanism

Microorganisms indirectly enhance soil carbon stocks through four principal mechanisms: (i) stimulating plant growth and nutrient acquisition, thereby increasing photosynthetic C allocation to aboveground biomass that ultimately enters soil as litter input; (ii) augmenting root exudation rates, which provide direct C inputs to SOC pools; (iii) improving soil aggregate stability; and (iv) mediating the chemical transformation of labile C compounds into soluble C [148]. PGPB and fungi serve as key drivers of these processes through their symbiotic relationships with vascular plants [150] (Fig. 3).

Key PGPB, including *Bacillus* spp., *Pseudomonas* spp., and *Rhizobium* spp., enhance plant productivity through two primary mechanisms: (i) modulation of root system architecture to improve acquisition of essential nutrients (C, N, and P) [40](as previously described) and (ii) production of bioactive compounds (e.g., antibiotics, extracellular enzymes) that mitigate abiotic and biotic stresses [151]. These synergistic effects lead to plants’ increased photosynthetic biomass production, whose subsequent incorporation into soil as plant litter significantly augments organic carbon stocks in paddy ecosystems. The hyphal diameter of AMF is much smaller than that of plant root hairs, enabling them to absorb nutrients from soil pores too small for root hairs. These nutrients are then transported to the roots, effectively expanding the root nutrient absorption zone and allowing plants to access a broader spectrum of soil nutrients. Weng et al*.* [46] summarized the primary mechanisms by which AMF-mediated interactions recruit various colonizing microorganisms. Their findings suggest that AMF hyphae can modify the permeability of host plant root cell membranes, leading to changes in the composition and quantity of root exudates released into the soil. As a result, the SOC pool is increased. Soil aggregates provide physical protection for SOC by shielding organic matter from microbial decomposition and oxidation. Furthermore, soil aggregates facilitate chemical adsorption by binding organic matter and ion compounds to the surface of clay particles, thereby reducing SOC loss and prolonging the residence time of carbon-containing compounds in the soil [152]. Microorganisms play a central role in soil aggregate formation and mineral adsorption [153]. Following experimental studies, Li et al*.* [154] found that AMF, through their symbiotic association with plant roots, can improve the quality of degraded soils and enhance soil aggregate stability.

### Factors influencing carbon storage in paddy soils

Factors limiting carbon storage in paddy soils can be broadly categorized into two groups: (i) agricultural management practices and (ii) natural climate variability.

#### Impacts of agricultural management practices

The impact of agricultural management on soil carbon stocks primarily involves the selection of fertilization methods, types, and application frequencies of organic fertilizers, along with tillage and other cultivation practices.

The application of chemical fertilizer alone has been shown to have low fertilizer use efficiency and a limited contribution to the SOC content in rice paddies. The degree of complementarity between organic and chemical fertilizers is an important factor influencing fertilization efficiency and organic carbon sequestration in paddy fields. Research indicates that, due to leaching, runoff, and emissions from the soil surface, less than 50% of applied chemical fertilizers are actually absorbed by plants [155]. Long-term application of fertilizers can also lead to soil acidification and crust formation, which in turn affect soil microbial abundance and functional activity [106, 156]. Jiang et al*.* [123] reported that applying nitrogen fertilizer at 225 kg N/ha was optimal for enhancing SOC sequestration and reducing greenhouse gas emissions while simultaneously achieving high rice yields. The use of organic fertilizers is considered a sustainable farming practice, as it can enhance soil carbon reservoirs and fertility [157]. However, due to factors such as nutrient concentration and release rate, the efficiency of applying organic fertilizer alone is relatively low. Consequently, substantial amounts of organic fertilizer, often measured in tons per hectare, must be applied to supply adequate nutrients for crops [158]. In recent years, the combined application of organic and inorganic fertilizers has become one of the fastest-growing areas of research in fertilizer utilization. Yang et al*.* [159], through long-term experiments, found that the combined use of organic and inorganic fertilizers can replace chemical fertilizer application to achieve high yields and significantly increase SOC content.

The types of organic fertilizers and their application frequency are also critical factors influencing carbon sequestration in rice fields. Different types of organic fertilizers contain varying amounts of organic matter and nutrients, and these differences determine their stability in soil and the rate at which they are degraded by microorganisms. An appropriate application frequency not only ensures sufficient nutrient availability for crops during critical growth stages but also effectively increases SOC. However, because rice paddy soils have a certain saturation threshold in their soil carbon pool, excessive application of organic fertilizers can lead to nutrient over-enrichment without significantly enhancing soil carbon storage [160]. Consequently, fertilization management should account for soil texture, mineral composition, and existing SOC/MAOC levels to assess the soil’s current carbon saturation stage. For paddy fields with substantial carbon saturation deficits, promoting the conversion of microbial residues to MAOM can be facilitated by combining organic fertilizer application with straw incorporation and optimizing water and nutrient management. On the other hand, for soils nearing their saturation threshold, the focus should shift toward improving nutrient use efficiency and the quality of carbon inputs, rather than merely increasing fertilizer quantities, to maximize the sequestration benefit from each unit of carbon added [161].

Cultivation and management strategies, including crop rotation systems and tillage methods, significantly impact soil carbon reservoirs in rice paddies. Agricultural intensification has ushered in discernible sustainability crises within agroecosystems, emphasizing the need to reevaluate the significance of diversified planting strategies—such as intercropping or crop rotation—under temporal and spatial considerations [180]. Consequently, soil management systems that maintain or enhance organic matter content are essential for increasing SOC levels [162]. Optimized rice paddy rotation practices can alter the soil environment, affecting the microbial community structure, diversity, and metabolites they produce in the carbon and nitrogen cycles, ultimately enhancing soil carbon storage [163, 164]. Variations in rotation patterns also significantly modify soil microbial community structure and enzyme activities related to carbon and nitrogen cycling [165]. Conventional tillage, rotary tillage, and no-tillage practices influence soil properties such as pH, bulk density, and soil organic carbon content. In turn, these changes affect the biological carbon sequestration process and the characteristics of soil microbial communities [166]. Traditional farming methods, characterized by chemical fertilizer application and frequent tillage, can achieve high rice yields but exhibit low SOC sequestration efficiency and significant greenhouse gas emissions. In contrast, practices such as no-tillage, crop rotation, and organic fertilizer application can enhance soil carbon sequestration. However, these methods may negatively impact rice yields. Therefore, optimizing the ratio of organic to inorganic fertilizer application (as discussed in Sect. "Effect of Fertilizer Application on Soil Fertility in Paddy") and integrating this strategy with practices such as straw return and conservation tillage is crucial for simultaneously achieving high rice yields and promoting soil carbon storage. In paddy fields, the interaction between fertilization and agricultural practices dictates SOC storage and rice yield. Practices such as straw incorporation and the replacement of chemical fertilizers with organic fertilizers have been documented to elevate rice yields [167] and, to a certain extent, foster SOC accumulation in paddy soils. Moreover, a study investigating the effects of agricultural management revealed that under the same fertilization, the combined practices of straw incorporation and 15 cm tillage significantly boosted both rice yields and SOC storage [168].

With rapid advancements in microbiome science, synthetic microbial communities (SynComs) are increasingly applied in agriculture, becoming another factor influencing the rice carbon pool. Through the bottom-up assembly and soil inoculation of microbial consortia with targeted functions [169], SynComs play a pivotal role in sequestering SOC. For instance, *Bacillus siamensi* and *Bradyrhizobium japonicum* can promote straw humification and enhance SOC content [170]. Furthermore, inoculation with SynComs, particularly those featuring cross-kingdom combinations of bacteria and AMF, significantly increases SOC levels in the rice rhizosphere [171]. This marks a paradigm shift in soil carbon management: moving from the regulation of abiotic environmental factors to the direct design and engineering of functional microbial consortia.

#### Impacts of natural climate change

Natural drivers predominantly include climatic variations and inherent soil properties. Climate change directly modulates organic carbon decomposition rates, thereby regulating microbial-mediated carbon transformation processes.

Climatic parameters (e.g., temperature, humidity) exert significant control over SOC formation and microbial activity. The study demonstrates that elevated temperatures enhance microbial necromass accumulation under fertilization conditions, thereby facilitating long-term soil carbon sequestration [172]. In humid tropical regions, fertilization promotes the association of microbial residues with mineral surfaces, forming MAOM [173]. Furthermore, temperature regulates microbial metabolic rates and extracellular enzyme kinetics, ultimately determining SOC decomposition efficiency [174]. Macro-level observations indicate that SOC content is generally lower in warm climate regions (e.g., subtropical and tropical) than in cool climate regions (e.g., temperate and warm temperate). Within the same climate zone, SOC content typically follows a decreasing trend: paddy fields > forests > upland fields [175]. While elevated atmospheric CO_2_ is expected to enhance net primary productivity and promote carbon sequestration in terrestrial ecosystems, the anticipated gains in soil carbon storage may be constrained by climate-driven increases in soil organic matter decomposition. As a result, the overall effect of global warming on grain yield is likely to remain modest, as losses in soil carbon pools partially offset the benefits of increased plant productivity [176]. In this context, “climate-smart fertilization”—an emerging strategy within the framework of low-carbon agriculture—offers a more adaptive pathway for the integrated application of organic and inorganic fertilizers. This concept has been substantiated by field trials in paddy soil ecosystems. For instance, studies indicate that the rational co-application of organic amendments (such as green manure and crop residues) with chemical nitrogen fertilizers can enhance soil aeration and stimulate functional microbial activity, thereby synergistically promoting soil carbon sequestration and climate change mitigation [177].

Soil environmental characteristics are additional limiting factors that influence microbial biomass and activity. Variations in soil properties across different soils exert differential effects on microbial functions, while microbial adaptability and metabolism directly impact the efficiency of ecological processes. Soil properties such as neutral pH, high clay content, elevated carbon content, and nutrient bioavailability facilitate plant growth, promote microbial biomass accumulation, and enhance the subsequent incorporation of OC into soil organic matter pools. Conversely, anoxic conditions tend to suppress microbial activity and slow microbial turnover rates, which can contribute to the accumulation of soil organic carbon [133].

From a soil type perspective, black soils exhibit the highest SOC content (up to 43.9 g/kg), significantly exceeding that of other soil types (by 41.6%−82.6%) [178]. Their outstanding carbon sequestration capacity may stem from a richer array of functional groups, higher ratios of iron-bound to total organic carbon (Fe-MIM/SOC), and a robust microbial potential for carbon sequestration driven by diverse bacterial and fungal communities [179]. Therefore, when assessing the mechanisms and potential for carbon storage at a specific scale, it is imperative to interpret these findings within the broader regional environmental context. Processes at different scales, from rhizosphere microsites to regional geographies, are nested and interact synergistically, collectively shaping the dynamics and ecological functions of rice soil carbon pools.

## Perspectives

Microbially-driven nutrient transformation in paddy soils and the mechanisms of nutrient uptake by rice provide a crucial theoretical basis for the sustainable management of paddy ecosystems. However, the complexity of this field means that numerous scientific questions remain to be addressed. Future research can explore and innovate in the following directions:

1. Elucidating the cascading response mechanism of fertilization patterns, microbial functions, and soil carbon–nitrogen cycles. Investigate the critical contribution and dynamic thresholds of specific microbial functional genes (e.g., genes related to methanogenesis and methane oxidation) that drive these cycles to soil carbon sequestration. Systematically track the dynamic characteristics of the fertilizer lifecycle carbon footprint and construct a quantitative coupling equation linking microbial function to carbon sequestration potential.
2. Integrating synthetic biology and materials engineering technologies to achieve synergistic innovation in microbial-driven fertilizer substitution and nutrient enhancement. Synthetic biology approaches for the rational design and targeted modification of rhizosphere probiotics or symbiotic nitrogen-fixation systems hold significant potential for constructing highly efficient, multifunctional genetically engineered microorganisms that enhance nutrient transformation efficiency. For example, genetic engineering enables the optimization of nitrogenase activity, thus establishing an in-situ microbial hub for nutrient cycling in the rhizosphere. Coupled with materials engineering, encapsulation and protection of microbial cells within microcapsules, hydrogels, or porous carriers can effectively mitigate soil biotic and abiotic stresses (e.g., pH fluctuations and water stress). Furthermore, integrating these engineered strains with smart responsive materials pre-loaded with slow-release fertilizers (e.g., pH-sensitive hydrogels triggered by root growth signals) creates a synergistic delivery system. This approach not only provides resilience against environmental stresses but also orchestrates microbial biological activation with physicochemical nutrient release, forming a precision-controlled cycle of signal-triggered microbial activity and nutrient supply.
3. Developing data-driven decision systems to promote the transformation of paddy field systems toward a smart, low-carbon model. Integrate real-time sensing, soil data (e.g., soil moisture, conductivity, temperature, dissolved oxygen) and microbiome data (microbial functional gene abundance information obtained from high-throughput metagenomic/metatranscriptomic analysis), combined with crop growth stages and nutrient demand dynamics, to construct a machine learning-based microbial environment crop collaborative response model. By establishing a dynamic fertilization decision support tool centered on the functional status of rhizosphere microorganisms, dynamic optimization of fertilizer supply strategies can be achieved.

## Data Availability

Not applicable.
